# Ca^2+^ entry units in a superfast fish muscle

**DOI:** 10.3389/fphys.2022.1036594

**Published:** 2022-10-28

**Authors:** J. Matthew Kittelberger, Clara Franzini-Armstrong, Simona Boncompagni

**Affiliations:** ^1^ Department of Biology, Gettysburg College, Gettysburg, PA, United States; ^2^ Marine Biological Laboratory, Woods Hole, MA, United States; ^3^ Department of Cell and Developmental Biology, University of Pennsylvania, Philadelphia, PA, United States; ^4^ Department of Neuroscience, Imaging and Clinical Sciences (DNICS), Center for Advanced Studies and Technology (CAST), University G. d' Annunzio (Ud'A) of Chieti-Pescara, Chieti, Italy

**Keywords:** electron microscopy, sarcoplasmic reticulum, store-operated Ca^2+^ entry, midshipman fish, sonic muscle, calcium homeostasis, excitation contraction coupling, vocal communication

## Abstract

Over the past two decades, mounting evidence has demonstrated that a mechanism known as store-operated Ca^2+^ entry (SOCE) plays a crucial role in sustaining skeletal muscle contractility by facilitating Ca^2+^ influx from the extracellular space during sarcoplasmic reticulum (SR) Ca^2+^ depletion. We recently demonstrated that, in exercised fast-twitch muscle from mice, the incidence of Ca^2+^ entry units (CEUs), newly described intracellular junctions between dead-end longitudinal transverse tubular (T-tubule) extensions and stacks of sarcoplasmic reticulum (SR) flat cisternae, strictly correlate with both the capability of fibers to maintain contractions during fatigue and enhanced Ca^2+^ influx *via* SOCE. Here, we tested the broader relevance of this result across vertebrates by searching for the presence of CEUs in the vocal muscles of a teleost fish adapted for extended, high-frequency activity. Specifically, we examined active vs. inactive superfast sonic muscles of plainfin midshipman (*Porichthys notatus*). Interestingly, muscles from actively humming territorial males had a much higher incidence of CEU SR stacks relative to territorial males that were not actively vocalizing, strengthening the concept that assembly of these structures is dynamic and use-dependent, as recently described in exercised muscles from mice. Our results support the hypothesis that CEUs represent a conserved mechanism, across vertebrates, for enabling high levels of repetitive muscle activity, and also provide new insights into the adaptive mechanisms underlying the unique properties of superfast midshipman sonic muscles.

## 1 Introduction

To maintain an adequate cytosolic Ca^2+^ concentration to support contraction and relaxation, skeletal muscle relies on a highly organized internal ultrastructure of proteins and membrane compartments. Ca^2+^ release units (CRUs), also called triads, are specialized intracellular junctions composed of a central transverse-tubule (T-tubule), an invagination of the sarcolemma membrane, flanked by two SR terminal cisternae or junctional SR (jSR) (Franzini-Armstrong and Jorgensen, 1994). CRUs are the site of excitation-contraction (EC) coupling, the mechanism that allows the transduction of the electrical signal coming from the motoneuron and depolarizing the exterior membranes (sarcolemma and transverse tubules or T-tubule), into Ca^2+^ release from the ryanodine receptors (RyRs), the calcium release channels of the SR (Schneider and Chandler, 1973).

In addition to the activation of EC coupling, over the past two decades, mounting evidence demonstrated that there is another mechanism, called store operated-Ca^2+^ entry (SOCE), that helps to maintain sustained contractility by facilitating the influx of external Ca^2+^ when the SR stores are depleted (Liu et al., 2005). In adult skeletal fibers from mice, we recently discovered and characterized exercise-dependent, intracellular junctions at the I band of sarcomeres which have been named Ca^2+^ entry units (CEUs). CEUs are proposed to enable rapid recovery of Ca^2+^ lost to the extracellular spaces during activity and thus to maintain sustained SR Ca^2+^ release and high-frequency, repetitive muscle contraction (Boncompagni et al., 2017, 2018; Michelucci et al., 2019, 2020). CEUs have a well-defined structural signature due to the association between stacks of multiple flat cisternae of SR origin and short, dead-end longitudinal extensions of the transverse tubular network, and in mouse muscles are exclusively located at the level of the I band. (Boncompagni, et al., 2017).

The function of CEUs is defined by the presence of the two core machinery proteins of the SOCE mechanism: STIM-1, the stromal interaction molecule-1 (STIM1) which acts as the Ca^2+^ sensor in the SR, and Orai1, a Ca^2+^ -permeable channel in the T-tubule (Boncompagni et al., 2017). Although previous works place STIM-Orai interaction at the triads, the sites of EC-coupling, CEUs provide a unique opportunity for close interaction between Orai1 T-tubule channels and STIM1 proteins in the SR. Indeed, small projections on the cytoplasmic surface of stack elements within CEUs (Boncompagni, et al., 2017) are also present in CEUs of other cells (Perni et al., 2015), and are tentatively suggested to be STIM1 molecules. These small projections are an additional identifying feature of CEUs, since such frequent tethers are not present in other SR areas. Presence of STIM1 at sites where CEUs are preferentially located has been recently confirmed (Zhang et al., 2021).

CEUs are not static organelles: while present at low incidence in muscles of resting mice (free to move about their cages), they are induced to assembly in significantly higher incidence in exercised muscles (Boncompagni, et al., 2017). Thus, CEUs are identified on the basis of specific criteria: their structural signature, their specific location, and their dependence on activity.

CEUs with these characteristics have been recently identified in mice (Boncompagni et al., 2017; Michelucci et al., 2019, Michelucci et al., 2020, Michelucci et al., 2022), and proposed as an exercise-dependent mechanism for SOCE, particularly to maintain sustained contractions of muscle fibers during fatigue. We tested this hypothesis by searching for the presence of CEUs in a completely different vertebrate muscle, adapted for extended, high-frequency activity. Specifically, we examined active vs inactive sonic muscles of plainfin midshipman (*Porichthys notatus*), a well-characterized and highly vocal species of teleost fish. Territorial male midshipman fish establish nesting sites under rocks in the intertidal zone during their breeding season. From these nests, males broadcast extremely long-duration (up to several hours; JMK, personal observation; Balebail and Sisneros, 2022), 100 Hz hums, by rapid contraction of a pair of sonic muscles located on the wall of their highly-inflated gas-filled swim bladders (Brantley and Bass, 1994; Bass, et al., 1999). The continuous contraction of these muscles creates a humming sound, with a 1:1 relationship between the frequency of muscle contraction and the fundamental frequency of the sound produced (a 100% duty cycle, Bass and Baker, 1990). The requirement for such extended, high frequency contraction necessitates specialized adaptations within the sonic muscles, to sustain both the energetics and Ca^2+^ dynamics of contraction (Rome, 2006). These adaptations are not fully understood, though it has been established that the superfast swim bladder muscles of territorial male midshipman have highly unusual morphological and physiological properties, that differ from the fast-acting sonic muscles of other, related, species of vocal fishes (e.g., the oyster toadfish, *Opsanus tau*), and of the less-vocal female and non-territorial male midshipman (Bass and Marchaterre, 1989; Brantley et al., 1993; Lewis et al., 2003; Nelson et al., 2018).

Here, we present evidence for the presence of numerous groupings of flat SR cisternae into multiple stacks with a preferred location opposite the border of the A band and I regions of the myofibrils in the superfast sonic muscles of territorial male midshipman fish. We tentatively identified the organelles as CEUs based on their morphology, and confirmed the identification on the basis of response to muscle activity: muscles from actively humming territorial males had a much higher incidence of these SR stacks relative to territorial males that were not actively vocalizing, similar to the findings in exercised vs. non-exercised mice (Boncompagni et al., 2017). This finding helps to strengthen the concept of a dynamic use-dependent assembly of these structures (Michelucci et al., 2019).

These results support the hypothesis that CEU SR stacks represent a conserved mechanism, across vertebrates, for enabling high levels of repetitive muscle activity, and also provide new insights into the adaptive mechanisms underlying the unique properties of superfast midshipman sonic muscles.

## 2 Methods

### 2.1 Fish: Housing, recording and selection of humming and not humming fish

Territorial male and gravid female midshipman fish were collected from nest sites in the intertidal zone in Bodega Bay, California (2016) or Seal Rock Beach, Washington (2019), during their breeding season in early June. Fish were shipped to the Marine Biological Laboratory in Woods Hole, MA, where they were housed in large (∼132 × 56 × 30 cm) tanks, with 4–6 males and 0–3 females per tank. Tank bottoms were covered with ∼3 cm of gravel substrate, and six artificial nest sites (∼30 cm diameter inverted ceramic flower pot saucers) were placed in each tank. Aerated, filtered flow-through seawater, at 12–15°C, was supplied to each tank. Fish were maintained on a 16:8 h light: dark cycle, and fed a diet of minnows (*Fundulus heteroclitus*). All animal care and use procedures were approved by the Marine Biological Laboratory’s Institutional Animal Care and Use Committee (protocol #’s 16–25 and 19–33).

Under these conditions, most (50%–80%) territorial male midshipman typically take up residence in a nest site, and begin producing their long-duration courtship “humming” vocalizations within 1–2 weeks of arrival in the lab. Once they begin, they hum nightly, for up to 8 h, for as long as about 4 weeks. Vocal behavior was monitored with hydrophones (Cetacean Research Technology model SQ26-MT) connected to digital recorders (Zoom model H1; input levels constant; sampling frequency = 44 kHz). Recordings were subsequently downloaded, and displayed and analyzed using Audacity software (v 3.0.2). When multiple fish in a tank were humming, individual humming fish were identified by moving the hydrophone closer to or further from different nest sites, and comparing relative amplitudes of the recorded hums, to determine the nest site(s) from which different fish were humming. After a putative humming fish was collected for perfusion (see below), recording was continued to confirm that humming from that nest site ceased. Furthermore, fish were confirmed to have been humming based on their dramatically inflated swim bladders; non-humming fish have obviously deflated bladders, and bladders deflate within about 24 h of the cessation of humming.

All fish identified here as “humming” were recorded to be humming immediately before muscle collection for perfusion; perfusion was complete within 30 min of last recorded humming in all cases. One fish (embedding identifier: PN19-003; as discussed below) had been humming regularly, but ceased humming 24 h before perfusion. This fish was defined as “humming,” as it still had a highly inflated swimbladder at the time of perfusion. Fish identified here as “non-humming” were never found to be humming during daily observations for at least 10 days prior to perfusion. “Non-humming” fish were nonetheless confirmed to be territorial because of their periodic or regular residence in a nest, and their body mass (typically 10-fold greater than that of the non-territorial male morph (Brantley and Bass, 1994) and other morphometric properties (see Section 3 and Section 4).

### 2.2 Perfusion and electron microscopy

For perfusion, fish were deeply anesthetized by immersion in 0.025% benzocaine in seawater. Morphometric measurements (body mass and length) were taken, and fish were then perfused transcardially with teleost Ringer’s solution followed by 4% paraformaldehyde in 0.1 M phosphate buffer, pH 7.2. The swimbladders with their attached muscles were dissected out and stored in 4% paraformaldehyde in 0.1 M phosphate buffer. Testes were also dissected out and weighed. Subsequently, a portion of each sonic muscle was dissected off of each excised bladder, post-fixed in 4% paraformaldehyde with 2.5% glutaraldehyde, and either used immediately for embedding, or shipped to Italy (to SB) for electron microscopy.

For E.M. embedding, the muscles were further post-fixed in 2% OsO4 in 0.1 M cacodylate buffer pH 7.2, washed in 0.1 M acetate buffer pH 5.4, *en-bloc* stained in 2% aqueous uranyl acetate for 1 h, dehydrated and embedded in Epon. Thin (∼80 nm) sections were cut using a Leica Ultracut R microtome (Leica Microsystem) with a Diatome diamond knife (Diatome Ltd.) and double-stained with uranyl acetate in 50% EtOH for 5 min and then 5 min in Sato’s lead solution.

### 2.3 Quantitative analyses of EM images

The number of flat-long stacks of cisternae at the I band of sarcomere per 100 μm^2^ area/section were determined from electron micrographs of non-overlapping regions randomly collected from transverse EM sections. For each specimen, three photographs taken at ×28,000 of magnification were obtained from sections of randomly selected areas of 5–10 different fibers. Only stacks of flattened and parallel series of cisternae with visible small electron dense strands apparently linking the cisternae were counted.

### 2.4 Statistical analyses

Statistical analyses were performed using JMP 8.0. Distributions were tested for normalcy using the Shapiro-Wilk test, and for equality of variance using Bartlett’s test. Standard t-tests were performed to compare data between groups when the data were normal and the variances equal. When variances were found to be unequal, a Welch test was used instead of a *t*-test; when distributions were not normal, a Wilcoxon test was used.

## 3 Results

### 3.1 Morphometric male midshipman fish parameters

In territorial male fish, we analyzed a number of morphometric parameters related to body size (standard length and body mass), condition (body mass index, or BMI, also known as Fulton’s K index, which equals: (body mass minus gonad mass) divided by the cube of the standard length; BMI is a standard indicator of body condition indirectly related to energy reserves in fish (Chellappa et al., 1995; Sutton et al., 2000; Bose et al., 2018)), and reproductive status (testes mass, and gonado-somatic index, or GSI, which equals testes mass expressed as a percent of body mass). All five parameters (Table 1) were not statistically different between humming and non-humming male fish.

**TABLE 1:** Humming (*N* = 5) and non-humming (*N* = 6) fish did not differ in size, body condition, or gonad size.

Morphometric parameter	Humming fish Mean±SD	Non-humming fish mean±SD	Statistical comparison
Standard Length (cm)	16.74 ± 1.35	18.82 ± 3.66	F (1,6.54) = 1.66, *p* > 0.24^a^
Body Mass (g)	63.8 ± 19.2	99.5 ± 63.8	F (1,6.06) = 1.70, *p* > 0.24^a^
Body Mass Index (BMI, g/cm3)	0.0131 ± 0.0006	0.0133 ± 0.0018	F (1,6.24) = 0.053, *p* > 0.82^a^
Testes Mass (g)	0.81 ± 0.42	0.89 ± 0.76	Z (32) = 0.27, *p* > 0.78^b^
Gonado-Somatic Index (GSI) (%)	1.24 ± 0.34	0.96 ± 0.56	t (9) = -0.95, *p* > 0.36^c^

^a^ Welch ANOVA; variances significantly unequal (*p* < 0.05) by Bartlett’s test.

^b^ Wilcoxon rank-sum; one or both distributions significantly non-normal (*p* < 0.05) by Shapiro-Wilk test.

^c^ Students’ *t*-test: variances equal and distributions normal.

### 3.2 Striking structural features of midshipman fish superfast muscles

**FIGURE 1:**
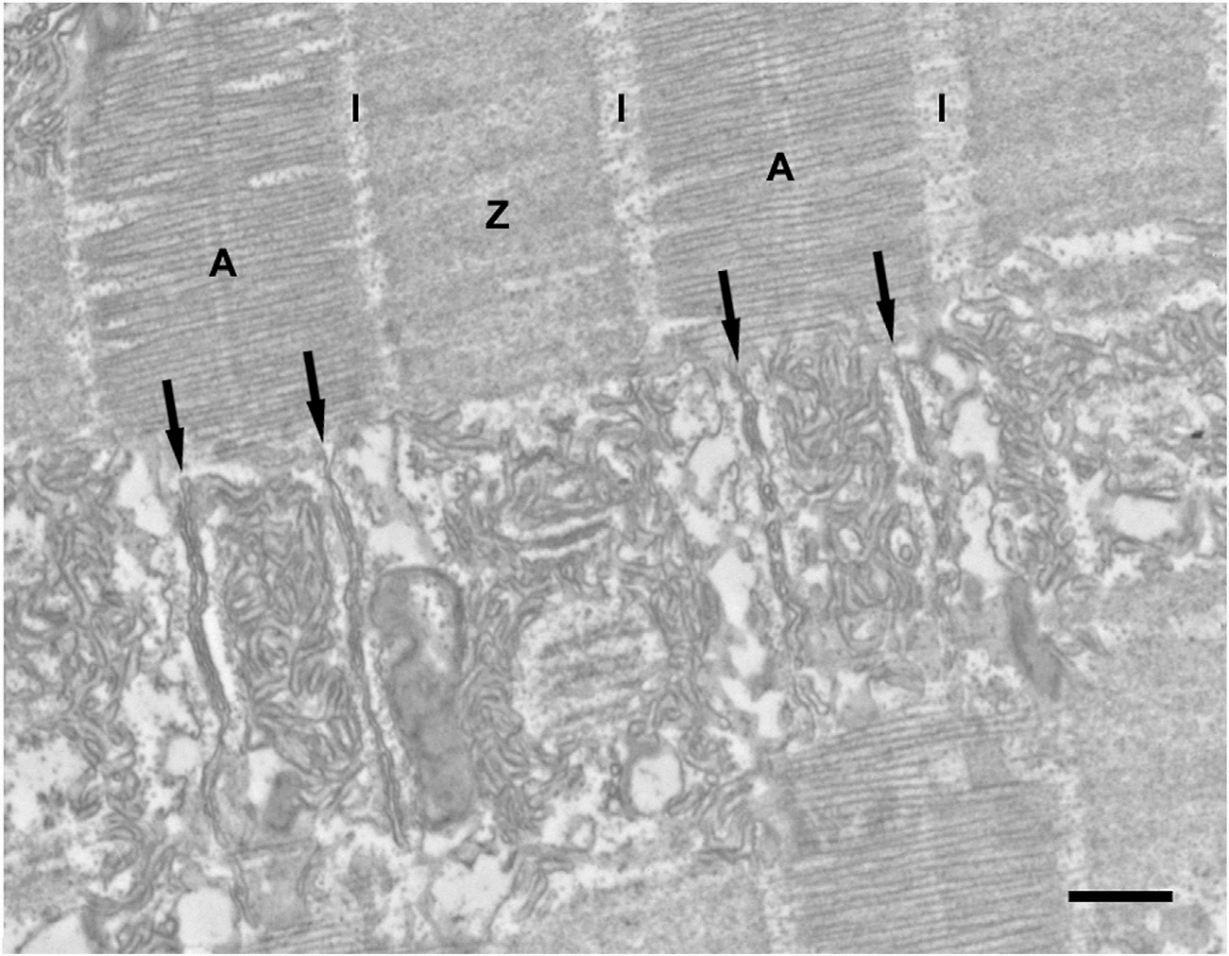
Representative EM image in longitudinal section of a sonic swimbladder muscle fiber from a territorial male midshipman. The upper part of the image shows the fiber cross striation. The A bands (A) alternate with Z bands (Z) of approximately equal width. The I band (I) is pale and very narrow because most of the thin filaments ovarlap with the thick filaments in the A band. In the lower half of the image, the thin section plane coincides with the layer of SR. In correspondence of each sarcomere, two triads (black arrows) are located at approximately one-third of the distance between the center and the edges of the A band. The SR forms an abundant convoluted network over the center of the A band and over the lateral sides of the sarcomere. Scale bars: 0.5 μm.

As in other fast acting sonic muscles (Fawcett, 1961; Revel, 1962), the myofibrils of midshipman fish swimbladder muscles are flat and ribbon like (Figure 1), which presumably allows rapid exchange of Ca^2+^ from its release sites at the triad to troponins. As others have noted previously (Bass and Marchaterre, 1989; Lewis, et al., 2003), mitochondria are segregated in the core and at the periphery of the fibers (not shown). The muscles have a highly unusual sarcomeric structure: standard A bands (A) alternate with short I bands and extended Z bands (Z) whose length equals that of the A band (Figure 1; Bass and Marchaterre, 1989; Lewis, et al., 2003). Since the thin filaments are excluded from the extensive Z lines, this reduces by approximately 50% the content of thin filaments and thus the amount of troponin that needs to be bound to Ca^2+^ for a full activation of the myofibril (see Section 4). The width of I bands, that is the region of the sarcomere that are composed only of thin filaments, depends on the muscle length at fixation and thus on the size of the swimbladder. Even in inflated swimbladders, after an active period of humming the I bands are narrow and most of the thin filaments’ lengths overlap the thick filaments in the A band. The I band is only slightly wider in muscles from actively humming fish, where the bladder is inflated (not shown).

There are two triads/sarcomere, each located at one-third of the distance between the center and edges of the A band (Figure 1, black arrows). This position is the same as in the toadfish swimbladder (e.g., see Appelt et al., 1991), but is distinctly different from the position of triads in other muscles (e.g., mammals), where the triads are located at the very edges of the A band and thus partly occupy an area opposite the I band.

**FIGURE 2:**
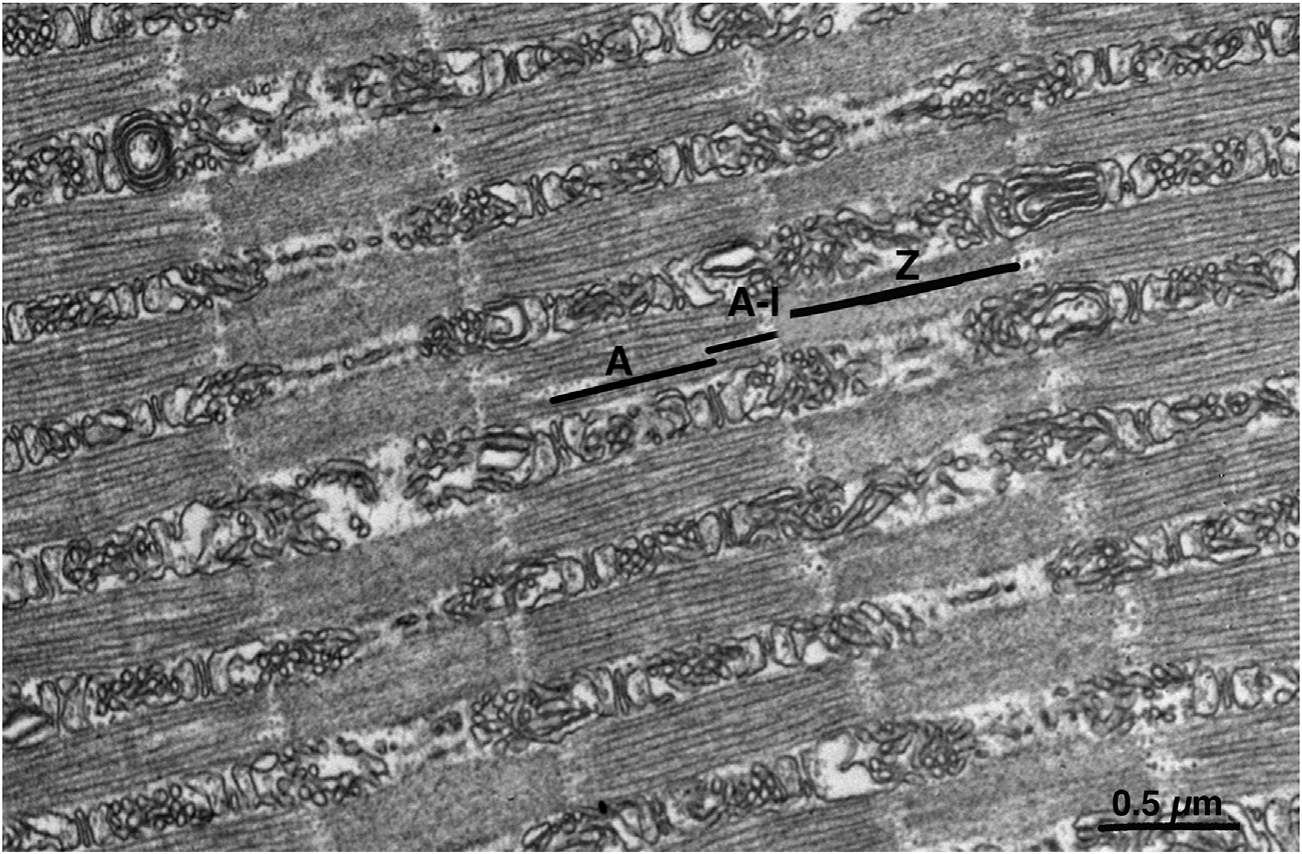
Representative EM image showing sarcomeric band regions in longitudinal section. Due to the position of triads within the A band region and to the extended Z lines (see Figure 1), the myofibrils’ surface is divided into three domains, each associated with defined portion of the sarcoplasmic reticulum network. One domain (A) covers the center regions of the A band between the triads. A second zone (A–I) covers the lateral thirds of the A band, the I band and the edges of the Z line. A third zone (Z) surrounds the myofibril at the level of the wide Z line. Z line. Scale bar: 0.5 μm.

The intermyofibrillar spaces are occupied by abundant SR elements divided into three segments. The A band SR (Figure 2) is associated with the myofibril surface limited by the positions of the two sets of triads. The SR at this level forms a double layer constituted of numerous tubules. The lateral edges of the A band and the adjacent narrow I band are the A-I zone (Figure 2). This region is occupied by numerous SR components continuous with the cisternae of the SR constituting the lateral domains of the A band triads. Near the ends of the Z line are groups of many short SR elements clustered together, while near the center of the Z line SR elements are scarce (Figure 2).

### 3.3 SR stacks: Unique SR configurations

**FIGURE 3:**
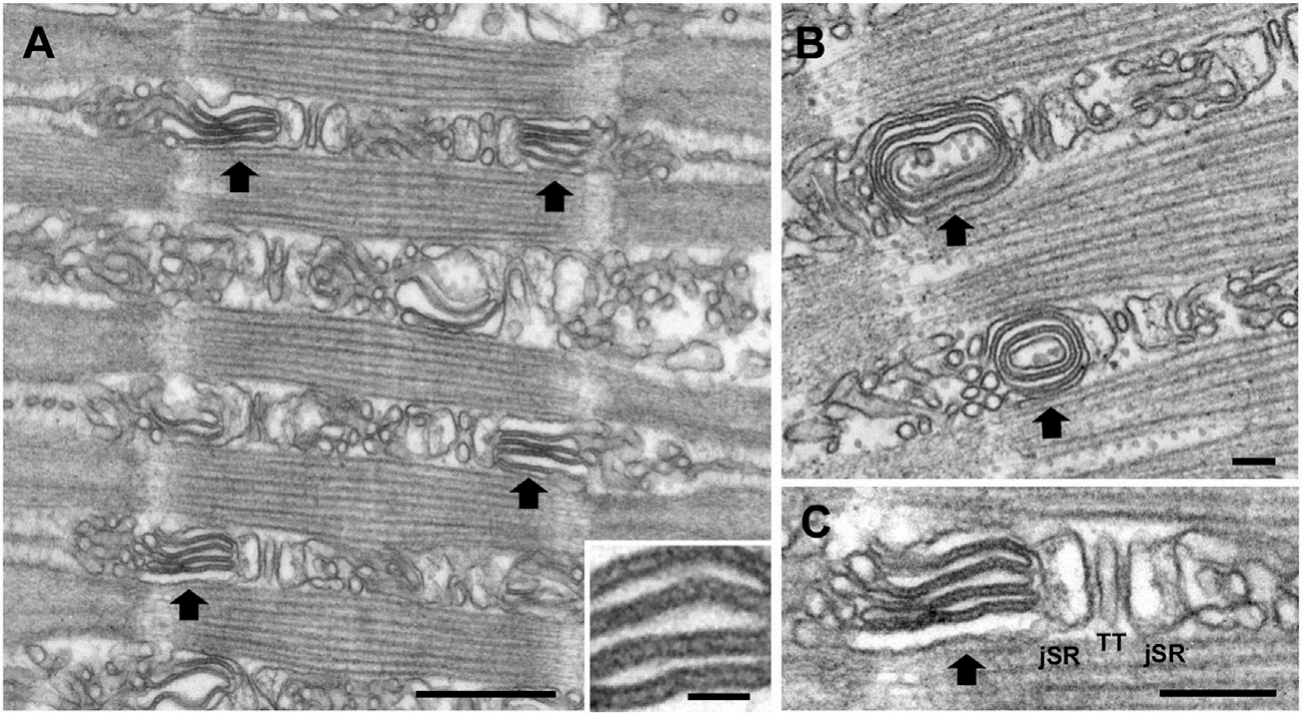
Representative EM images of sonic muscle fibers from a humming male midshipman, showing SR stacks. At random intervals within the fibers, the intermyofibrillar spaces are occupied by flat SR cisternae arranged into tight stacks (large black arrows) forming either flat aggregates **(A)** or concentric circles **(B)**. The stack components are in direct continuity with the SR elements (jSR) constituting the lateral sacs of the triad **(C)** (TT i.e., T-tubule). The spacing between the surfaces of cisternae is apparently constant and crossed by frequent links or tethers (inset in A). The stacks are found in the sonic swimbladder muscles of all territorial male fish, but more frequently in muscles that have been used for an extended period of humming, as in the fish depicted here (see also Table 2). Scale bars: 0.5 μm **(A)**, 0.05 μm (inset), 0.1 μm **(B)**, 0.2 μm **(C)**.

In the A-I region, the lateral sacs constituting the peripheral side of the junctional triadic SR are on occasion morphed into elongated flat elements closely apposed to each other (the “stacks”), often, but not always, in circular forms (Figure 3B). The stacks of cisternae occupy the entire A-I region and their components are clearly continuous with the SR elements of the adjacent triadic cisternae (Figure 3). Three details indicate the uniqueness of the stacks. First, the stacks are always in continuity with the SR composing the lateral sacs on the peripheral side of the triads, i.e., junctional SR (jSR) (Figure 3C) and only the SR lateral sacs facing the edges of the sarcomere and not those from the triad side facing the center of the A band are involved in forming stacks. For this reason, the stacks are never located opposite the A band region of the sarcomere, but are always positioned opposite the lateral third of the A band, in the intermyofibrillar spaces region identified as A-I in Figure 2. This is a region equivalent to the I band of sarcomeres in other muscles (e.g., from mouse) where the triads are located at the very edges of the A band. No stacks are found opposite either the A band or in the region opposite the Z lines. Second, the stack cisternae are of fairly constant width and closely apposed to each other, forming either concentric circles (Figure 3B) or flat parallel aggregates (Figure 3C). Third, the spacing between the surfaces of cisternae is constant and crossed by frequent links or tethers (Figure 3A, inset). Such short tethers are not present on the surface of other SR elements. Four to five elements are involved in each stack. The arrangement is quite distinct from that of the remaining SR, which is composed of randomly arranged sacs and cisternae in variable, sometimes close, proximity to each other but with variable shapes, width and distances and no tethering components. Thus, the stacks are unique, well defined SR components.

The stacks are not evenly distributed within the fiber and their incidence is variable even within the same specimen [note below the large standard deviation (SD) of data in Table 2]. Variations in incidence between and within muscles fibers indicate that stacks are not apparently fixed components of the fibers, such as the triads, but components that may form and dissociate over time. This is confirmed by the EM quantitative analysis of stacks incidence (see data below).

**TABLE 2:** SR stack incidence in sonic muscles from humming (*N* = 5) vs. non-humming (*N* = 6) fish.

Fish/embedding identifier	Stack incidence (no. per 100 μm^2^) Mean±SD	Humming status	Time from last observed humming to perfusion
PN16-001/6125	10.3 ± 18.9	Not humming	>10 days (never observed humming)
PN16-002/6126	16.6 ± 19.2	Not humming	>14 days (never observed humming)
PN16-003/6127	28.0 ± 21.9	Not humming	>17 days (never observed humming)
PN16-010/3210	15.5 ± 22.3	Not humming	>32 days
PN16-011/3211	27.7 ± 17.6	Not humming	22 days
PN19-002/3213	16.5 ± 16.2	Not humming	>21 days (never observed humming)
PN16-007/6128	40.4 ± 28.7	Humming	<0.5 h
PN16-008/6129	40.4 ± 22.5	Humming	<0.5 h
PN16-009/6130	54.8 ± 25.6	Humming	<0.5 h
PN16-013/3212	34.2 ± 20.4	Humming	<0.5 h
PN19-003/3214	30.6 ± 18.0	Humming	1 day

### 3.4 Active sonic muscle fibers exhibited higher incidence of SR stacks compared to non-active male midshipman fish

Based on their structural signature and location, the stacks can be tentatively identified as CEUs (see Section 4). If this identification is correct, the stacks’ incidence should be affected by muscle usage, as is the incidence of CEUs in exercising mice (Boncompagni et al., 2017). This was tested by stereology as follows. Sonic swimbladder muscles from “resting” and active fish (i.e., from non-humming and humming fish, respectively) were obtained as indicated in the Methods section. The relative incidence of SR stacks was estimated by counting the number of stack profiles per unit area in cross sections of muscle from random images (see Section 2). The ratio of stacks to section area is directly proportional to the ratio of stacks to fiber volume. The ratio is affected by the sarcomere length at which the muscles were fixed, but since this varied by very little in different specimens (not shown), no correction was applied. Stacks were counted based on the structural criteria defined above (flat, parallel cisternae with visible small electron dense strands; see also Figure 3). The data are presented as the ratio of stacks per unit area in Table 2 (as in Boncompagni et al., 2017).

Fish that had been humming immediately or shortly prior to muscle collection exhibited a 2-fold higher incidence of SR stacks, on average, in their sonic swimbladder muscles than fish that had not been humming for at least 10 days (Table 2; mean of 40.1±9.2 stacks per 100 μm^2^ in humming fish vs. mean of 19.1±7.2 stacks per 100 μm^2^ in non-humming fish). The difference in stack incidence between humming and non-humming fish was statistically significant (Students’ *t*-test: t (9) = −4.25; *p* < 0.003). The ranges of stack incidence for humming and non-humming fish were non-overlapping: the greatest observed incidence for non-humming fish (28.0) was less than the lowest observed incidence for humming fish (30.6). Interestingly, the one fish (PN 19–003) that had ceased humming shortly (24 h) prior to collection, and still had a highly inflated bladder had a relatively high content of stacks, though lower than the incidence of other actively humming fish (see Section 4). Stacks did remain present, albeit at lower incidence, in “resting” sonic muscles from non-humming fish, even after a delay of as long as a month after the fish ceased humming. Among non-humming fish, there was no statistically significant relationship between stack incidence and the minimum number of days since the fish last hummed [ANOVA, F (1,5) = 0.081, *p* > 0.79].

## 4 Discussion

We examined sonic muscles in territorial male midshipman, not in the non-vocal and non-territorial “Type II” male morph. Only territorial males produce courtship hums (Brantley and Bass, 1994). We are confident that non-humming males in our study were nonetheless territorial based on three criteria: 1) body mass, > 40 g, was at least double the known mass of the largest Type II males (Bass and Marchaterre, 1989; Brantley, et al., 1993; Brantley and Bass, 1994), and was no different from our humming male population; 2) gonadosomatic index, which was ∼1% of body mass, in both humming and non-humming males, was similar to previously published reports of GSI in territorial males, and was about 10-fold less than GSI in Type II males (Bass and Marchaterre, 1989; Brantley and Bass, 1994); and 3) behavior, as some of our non-humming males were observed to be humming at some point, and many at least periodically took up residence in a nest, behaviors not observed in Type II males.

Individual territorial male midshipman remain in their nests, courting females and guarding eggs, for up to about 60 days in the wild (Brantley and Bass, 1994). In captivity, under the housing conditions used here, individual males will hum nightly for up to about a month before ceasing (JMK, personal observations). Territorial males are thought to eat very little while defending nests, both in the wild and in captivity (Cogliati et al., 2013; JMK personal observations). Thus, it is possible that fish stop humming due to a decrease in energy reserves, and that an important difference between our non-humming fish and our humming fish, that could be related to the differences in SR stack counts, could thus be a difference in overall body condition. However, the fact that body mass index did not differ between our humming and non-humming fish argues against this possibility. Furthermore, because gonadosomatic index did not differ between humming and non-humming males, and was consistent with GSI values in breeding territorial males from other studies, the known seasonal decline in testes size (and also testosterone levels) occurring after the end of the breeding season (Sisneros et al., 2004) could not have been a factor influencing the observed differences in SR stack incidence between the two groups. We therefore conclude that the increased incidence of SR stacks in sonic muscles is directly correlated with muscle activity occurring during humming, rather than to some other aspect of the fish’s condition.

Stacks of SR cisternae in the sonic muscles of the midshipman fish fit the four criteria that identify Ca^2+^ Entry Units (CEUs) in mouse muscles: 1) configuration of SR into multiple flat, parallel cisternae linked by visible tethers; 2) continuity of stack elements with the jSR constituting the peripheral side of triads; 3) specific location of the stacks at the A-I region (see description above); and 4) increased incidence of SR stack elements with muscle exercise. An additional structural detail, the presence of extensions of the T tubule network into the stacks, is not shown in these data, because it requires a structural technique that was not applied in this work (Michelucci et al., 2019). In addition, a limitation of this study is that we did not directly demonstrate the presence of the two molecular players of SOCE (STIM1 and Orai1) in SR stacks and T-tubules, respectively. Future studies designed to monitor STIM1 And Orai1 specifically within the “CEUs” in midshipman will be needed.

However, for the reasons listed above, it is reasonable to assume that the SR stacks we describe here in midshipman swimbladder muscles are indeed functional CEUs, involved in the replenishment of intracellular Ca^2+^ lost during the period of prolonged activity of a hum. This identification of presumptive CEUs in midshipman sonic muscles shows that CEUs are physiological components of muscle fibers at a considerable phylogenetic distance from mammals, where they were first observed, and thus are common, plastic organelles of skeletal muscles, enabling adaptation to prolonged periods of high activity.

The sonic muscles in midshipman swimbladder are fully and continuously active, contracting at frequencies of ∼100 Hz for well over 1 h at a time during episodes of courtship “humming”. When electrically stimulated in the lab, midshipman sonic muscles are unable to contract at higher frequencies (Nelson, et al., 2018), suggesting that they are operating at or near their physiological maximum. Midshipman vocalizations are longer than those of any other known vertebrate, providing a unique opportunity to study how muscles adapt to sustain high frequency contractions over extended time periods. Two major functional and structural adaptations have been identified as the basis of the extraordinary behavior of the midshipman muscles. One is the very small size of the Ca^2+^ transients, which minimizes both the energy required for Ca^2+^ cycling, and the amount of Ca^2+^ that needs to be pumped back into the SR in the ∼10 msec before the next contraction (Nelson et al., 2018). The second is the structural adaptation of the extended Z lines (Bass and Marchaterre, 1989; Lewis, et al., 2003) that reduces by approximately 50% the content of troponin that needs to be activated by Ca^2+^. Since midshipman sonic muscles have relatively low levels of Ca^2+^-buffering parvalbumin (Tikunov and Rome, 2009), equilibrium requires that Ca^2+^ is fully recycled into the SR at the end of each humming cycle (Nelson et al., 2018). The cytoplasmic free Ca^2+^ level, although reduced, is higher than in the toadfish muscle (Nelson et al., 2018) and it is expected that some leak of Ca^2+^ to the outside may occur during each humming period, requiring a complementary Ca^2+^ entry mechanism, to sustain SR Ca^2+^ concentrations over the hours-long duration of a hum, through the SOCE machinery. This study demonstrates an additional specialization of active midshipman sonic muscle fibers: the presence and activity-related induction of organelles, i.e., CEUs, that are presumably dedicated to the function of maintaining Ca^2+^ equilibrium over the duration of an hours-long hum.

Our experimental data allow some limited conclusions about the rate of decay of exercise-induced SR stack incidence after muscle activity ends. Our non-humming fish ceased humming between at least 10 and at least 32 days prior to muscle collection. Over this time period, we observed no statistically significant correlation between SR stacks incidence and days since the last possible humming. Furthermore, we noted that the one humming fish (PN19-003) that had ceased humming 24 h prior to muscle collection had the lowest SR stack incidence of any humming fish, only slightly above the highest incidence observed in non-humming fish. It therefore appears that SR stacks begin to decrease in incidence within 24 h after fish stop humming (similar to the time course of swimbladder deflation), reaching an asymptotic baseline level after sometime between 1 and 10 days. Therefore, SR stacks rapidly remodel after sustained muscle activity ends. This is consistent with data reported for exercise-induced assembly of CEUs these SR/T-in extensor digitorum longus (EDL) fast-twitch muscle in mice, as they increase during acute exercise and then progressively disassemble following a subsequent period of inactivity (≥6 h) (Michelucci et al., 2019).

Previous to this study, SR stacks, identified here as putative CEUs based on comparison with studies in mice (Boncompagni, et al., 2017), had not been noted in publications examining midshipman sonic muscles by EM (e.g., Bass and Marchaterre, 1989; Lewis et al., 2003). Lack of previous observations is likely due to the fact that CEUs were not identified until recently and thus easily escaped attention as worthy of note. Furthermore, as we describe here, these structures are significantly upregulated by humming activity, and previous studies did not use actively humming fish. We were also surprised to note that stacks (or CEUs) have never been reported in the sonic muscles of *Opsanus tau* (oyster toadfish), despite the fact that the muscle has been the subject of numerous investigations by electron microscopy, included several by Prof. Clara Franzini-Armstrong’s laboratory (Franzini-Armstrong and Nunzi, 1983; Ferguson et al., 1984; Block et al., 1988; Appelt et al., 1991). A careful re-examination of a large number of samples, based on our current knowledge of CEUs identifying features, confirms this absence (data not shown). Since the vocal behavior status of the toadfish used in the previously published studies was not checked, we cannot confirm the effect, if any, of activity on CEU incidence in these muscles. However, we can conclude that, unlike midshipman sonic muscles, where CEU’s appear present even in the baseline, resting, state, comparable CEU’s are not present in resting sonic muscles of *Opsanus tau*. The mating calls of toadfish are fundamentally different from those in midshipman: they are periodic, as the vibration is maintained for only for 200–300 msec and then stops for 5–15 s before the next call, providing the muscle time to re-charge (Fine et al., 1977). Although the small Ca^2+^ transients in toadfish are larger than in the midshipman, the cytoplasmic concentration of Ca^2+^ remains low due to the presence of parvalbumin, a protein with high affinity for Ca^2+^ that effectively binds all the free Ca^2+^ (Tikunov and Rome, 2009; Nelson et al., 2014; Nelson et al., 2018.) Thus, presumably, little or no Ca^2+^ is lost outside the fibers during calling. Equilibrium is reached during the recovery period between calls, when Ca^2+^ is slowly released from parvalbumin and pumped back into the SR. Essentially, Ca^2+^ cycling in toadfish sonic muscles may not involve the extracellular space and thus the muscle activity does not require CEUs. Comparison of the sonic muscles in toadfish and midshipman may thus illustrate the concept that CEUs presence and incidence are controlled by cellular SOCE requirements imposed by the vocal behavior itself.

Finally, in the control of skeletal muscle Ca^2+^ homeostasis, a pivotal role is also played by the mitochondria as they take up Ca^2+^ in response to cytosolic [Ca^2+^] increases for stimulating oxidative metabolism (Rudolf et al., 2004). In midshipman fish fibers, the numerous mitochondria necessary to sustain energy consumption for fast Ca^2+^ pumping are segregated in the core and at the periphery of the fiber (Bass and Marchaterre, 1989; Lewis et al., 2003), and are not mixed with myofibrils and SR as they are in mammalian muscles (not shown; Boncompagni et al., 2009). However, investigation of the possible role of mitochondria Ca^2+^ uptake in the regulation of Ca^2+^ homeostasis in midshipman vocal muscle fibers is beyond the scope of the current study.

## 5 Conclusion

The results presented in this study strengthen the concept that CEUs are intracellular structures whose incidence strictly correlates with intense/sustained muscle activity. As for EDL (extensor digitorum longus) muscles from exercised mice, also in superfast midshipman sonic muscles from humming fish, we found a high incidence of SR stacks structurally identical to SR stacks described in CEUs as sites involved in SOCE (see Protasi et al., 2021 for a review).

Worthy of note is that, as clearly appears in EM images, within a CEU, the SR stacks’ lumen is continuous with the jSR lumen of a triad. In addition, in a recent elegant and careful work by Lavorato et al., 2019, it has been described that the whole SR lumen is continuous throughout an entire muscle fiber. Our works, in exercised mouse muscles (Boncompagni et al., 2017) and, here, in actively humming fish, showed a higher incidence of CEUs, and thus of SR stack elements, which, at least in mice, contain STIM1 proteins, in fibers that experience sustained/repetitive muscle contraction. Therefore, it is reasonable to think that SR stacks could create a preferential pathway for extracellular Ca^2+^ to refill nearby jSR needed to maintain SR Ca^2+^ release, when SR Ca^2+^ content and/or buffering capacity may be reduced, as also occurs in mice that are deficient of the SR Ca^2+^ buffer proteins, calsequestrins (Michelucci et al., 2022).

These results may help explain how superfast midshipman sonic muscles are adapted to sustain the long duration, high frequency contractions necessary for production of courtship vocalizations in this species. An additional important finding of this study is that CEUs may represent a conserved evolutionary mechanism for enabling high levels of repetitive muscle activity across vertebrates.

## Data Availability

The original contributions presented in the study are included in the article/supplementary material, further inquiries can be directed to the corresponding author.

## Abbreviations

CEU, Ca^2+^ entry unit; CRU, Ca^2+^ release unit; EC coupling, excitation-contraction coupling; EM, electron microscopy; SR, sarcoplasmic reticulum; SOCE, store-operated Ca^2+^ entry; STIM1, stromal interacting molecule-1; T-tubule, transverse tubule.
